# Role of regional anesthesia in minimizing opioid use and enhancing patient recovery: a case series

**DOI:** 10.1186/s13256-025-05177-3

**Published:** 2025-04-16

**Authors:** Irwan Setiadi, Muhammad Rezanda Alifahna, Radian Ahmad Halimi, Dewi Yulianti Bisri

**Affiliations:** Department of Anaesthesiology and Intensive Therapy, Faculty of Medicine, Universitas Padjadjaran/Dr. Hasan Sadikin General Hospital, Pasteur Street 38, Bandung, West Java 40161 Indonesia

**Keywords:** Regional anesthesia, ERAS, Spinal surgery, Anesthesia, Case series

## Abstract

**Background:**

Enhanced Recovery After Surgery employs evidence-based strategies to improve outcomes across the preoperative, intraoperative, and postoperative phases. In spinal surgery, compared with traditional management with general anesthesia, regional anesthesia offers benefits such as reduced pain, improved organ function, faster mobility, and shorter hospital stays. Studies have shown that regional anesthesia, particularly spinal anesthesia, results in shorter operation times, lower postoperative pain, and fewer thrombosis complications than does general anesthesia. The implementation of regional anesthesia in Enhanced Recovery After Surgery protocols for elective spinal surgeries aims to minimize hospital stays and opioid use, significantly enhancing recovery and patient outcomes.

**Patient presentation:**

Two Sundanese patients underwent laminectomy surgery for chronic low back pain and disc degeneration. The first, a 57-year-old man, presented with bulging discs and osteophytes in the lumbar spine and underwent a 2-hour surgery in the prone position. His vital signs remained stable throughout. The second patient, a 54-year-old woman, also had similar lumbar spine issues and underwent equally successful surgery under stable hemodynamic conditions.

**Conclusion:**

Regional anesthesia during lumbar spine surgery ensures stable perioperative hemodynamics and reduces opioid needs, which aligns with Enhanced Recovery After Surgery principles, promoting faster recovery and better outcomes.

## Introduction

Enhanced Recovery After Surgery (ERAS) is a multidisciplinary approach to improve patient care via evidence-based methods. The ERAS protocol can be divided into preoperative, intraoperative, and postoperative management. The ERAS protocol has been carried out in a variety of surgical procedures, one of which is spinal surgery. Spinal surgery is one of the most painful surgical procedures, thus postoperative pain control is challenging and requires additional opioid drugs in the perioperative period [1]. Compared with regional anesthesia, general anesthesia is more commonly used in spinal surgery. Regional anesthesia may be an option if the patient has multiple comorbidities or is elderly. Regional anesthesia has generally been shown to reduce pain, improve organ function, increase mobility, reduce the incidence of nausea and vomiting, and reduce the length of hospital stay. Several studies comparing general anesthesia with regional anesthesia, especially spinal anesthesia, for spinal surgery have shown shorter operating times, lower postoperative pain, shorter times in the postanesthesia care unit (PACU), lower incidences of urinary retention and postoperative nausea, and favorable cost-effectiveness [2, 3]. Previous studies have shown that deep vein thrombosis is more common in patients receiving general anesthesia than in those receiving regional anesthesia. A reduction in thromboembolic complications has also been reported in spinal surgery patients receiving regional anesthesia [4]. This case report was conducted as part of our process to implement the ERAS protocol by implementing regional anesthesia for elective spinal surgery procedures at our institution so that quality improvement can reduce the length of stay and opioid requirements in the hospital [2, 3].

## Case series

The procedure was explained to the patients and they agreed that the their data could be used as a case report and published. Our hospital ethics committee approved the collection of patient data to be processed into a scientific report and published.

### Case I

**Fig. 1:**
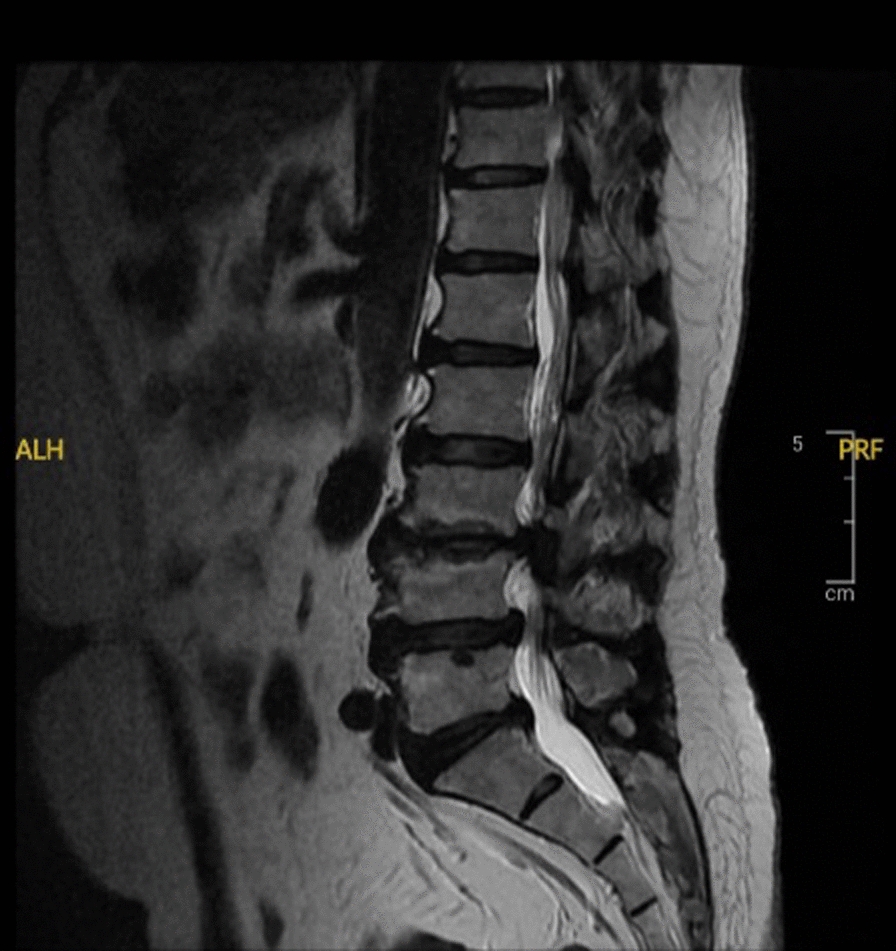
Lumbosacral magnetic resonance imaging of patient I

A 57-year-old Sundanese man weighing 65 kg (kg) with a height of 168 cm suffered from lower back pain and tingling for the past 2–3 years. There was no history of illness or disorder in the patient’s family and psychosocial background. On examination, the general condition included a blood pressure of 120/80 mm of mercury (mmHg), a heart rate of 80 beats per minute, a respiration rate of 18 breaths per minute, and oxygen saturation of 98%. The laboratory results and thorax X-ray results were within normal limits. Magnetic resonance imaging (MRI) revealed bulging intervertebral discs of lumbar vertebrae 4–5 and lumbar vertebra 5–sacral vertebra 1; osteophytes on the endplates of lumbar vertebrae 3, 4, 5; and degeneration of the discus intervertebral vertebrae 4–5 and lumbar vertebra 5–sacral vertebra 1 (Fig. 1). The patient was scheduled for laminectomy surgery. The surgery was performed while the patients were in the prone position and lasted 2 hours. The patient was monitored for blood pressure, pulse rate, respiratory rate, and saturation during surgery and was found to be hemodynamically stable.

### Case II

**Fig. 2:**
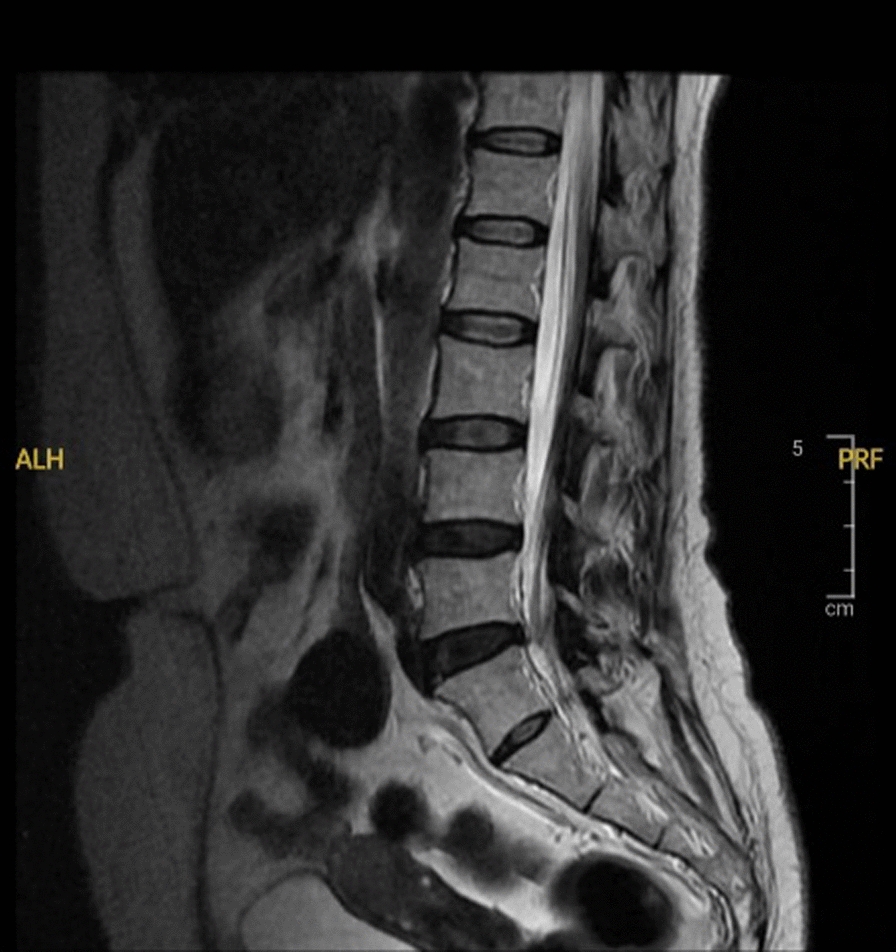
Lumbosacral magnetic resonance imaging of patient II

A 54-year-old Sundanese woman weighing 50 kg with a height of 159 cm experienced worsening back pain for the last year. There was no history of illness or disorder in the patient’s family and psychosocial background. On examination, the patient’s general condition included a blood pressure of 115/70 mmHg, heart rate of 68 beats per minute, respiration rate of 18 breaths per minute, and saturation of 99%. The laboratory results and thorax X-ray results were within normal limits. MRI revealed bulging intervertebral discs of lumbar vertebrae 4–5 and lumbar vertebra 5–sacral vertebra 1; osteophytes on the endplates of lumbar vertebrae 3, 4, and 5; and degeneration of the intervertebral discs of lumbar vertebrae 4–5 and lumbar vertebra 5–sacral vertebra 1 (Fig. 2). The patient was scheduled for laminectomy surgery. The surgery was performed while the patients were in the prone position and lasted 2 hours. The patient was monitored for blood pressure, pulse rate, respiratory rate, and saturation during the operation and was found to be hemodynamically stable.

### Anesthesia management

The regional anesthesia plan and informed consent regarding the scientific publication of the case series were explained to the patient. The patient was given 6 hours of heavy food and 2 hours of high-carbohydrate clear drink. The patient was given 1000 mg (mg) of paracetamol orally and 300 mg of gabapentin orally in the morning.

The patient was given an infusion of lactated Ringer’s solution of 300–500 milliliters (mL) 10–15 minutes before spinal anesthesia started. Upon arrival at the operating room, the patient was placed in a seated position. After local infiltration with 2–3 mL of 2% lidocaine, spinal anesthesia was performed via a 25 G needle. The spinal needle was inserted at third–fourth lumbar level. Bupivacaine 0.5% 12.5 mg was administered along with fentanyl 25 mcg. After the anesthetic agents were administered, the patient was placed in a prone position on the operating table. Oxygen was administered via a nasal cannula, and light sedation via midazolam was given intermittently.

After completion of the procedure, patients were taken to the PACU for recovery until they achieved adequate motor function in the lower extremities, followed by transfer to the treatment room. Patients were given paracetamol (1 g every 6 hours intravenously), gabapentin (300 mg every 24 hours), and tramadol (50 mg every 12 hours). Both patients were able to mobilize and start oral intake 6 hours postoperatively. Patients began to do light moves independently 12 hours postoperatively. Patients have been able to move independently without any pain 24 hours postoperatively. On the second postoperative day, both patients were discharged.

## Discussion

Spinal surgery can be performed with various anesthetic techniques; however, the use of regional anesthetic techniques, such as spinal anesthesia, has been widely recommended, especially for lumbar spine surgery, such as discectomy and laminectomy. Spinal anesthesia has several benefits, including rapid onset, decreased intraoperative blood loss, thrombotic events, pulmonary complications and postoperative dysfunction. It has been suggested that the reduction in intraoperative blood loss may be due to a decrease in heart rate and mean arterial pressure (MAP) from sympathetic blockade. In a retrospective study of 544 patients who underwent spinal surgery, spinal anesthesia was associated with a shorter operating time, shorter intraoperative blood loss, and a shorter duration of hospital stay [3].

A recent study evaluated the risks of general anesthesia in elderly patients undergoing spinal surgery. Deyo *et al*. reported that 3.1% of complications, including cardiac arrest, acute myocardial infarction, respiratory failure, pulmonary embolism, pneumonia, and stroke, occurred. Patients under regional anesthesia are less prone to hypoxemia than are those under general anesthesia. Recent studies have suggested that elderly patients with American Society of Anesthesiologists (ASA) physical classification status grades III and IV have a high risk of general anesthesia and therefore suggest the use of spinal anesthesia techniques [4]. This study confirms the results of previous studies, which showed that spinal anesthesia is a safe and effective technique for lumbar spine surgery. Although there was no significant difference in morphine consumption in the first 48 hours after surgery between regional anesthesia and general anesthesia in this study, regional anesthesia was strongly associated with lower pain scores during the PACU stay, shorter anesthesia times, and higher levels of patient satisfaction than was general anesthesia [5].

In this case series, before surgery, the patient was fed 6 hours of heavy food and 2 hours of high-carbohydrate drinks. During the preoperative period, prolonged fasting should be avoided, as it has been shown to have negative side effects on muscle catabolism. Patients can eat up to 6 hours before surgery and can drink clear liquids up to 2 hours before surgery, and carbohydrate supplementation is recommended. The ERAS protocol for spinal surgery focuses on minimizing anesthetic agents with short sedatives. The nutritional component of perioperative care includes clear carbohydrate fluids starting from 1 day to 2 hours before surgery and a return to a regular postoperative diet on the day of surgery if possible [1, 6, 7].

All patients in this case series received 1000 mg of acetaminophen and 300 mg of gabapentin orally on the morning of surgery as one of the implementations of the ERAS protocol before surgery. The preoperative components of the ERAS protocol include preemptive analgesia (for example, gabapentin/pregabalin, acetaminophen), nutritional optimization and fasting, and patient education. The ERAS protocol, with its synergistic multimodal anesthetic strategy, is focused on avoiding the negative side effects of opioids. The reported preoperative treatments include 1 g of intravenous acetaminophen, a 600 mg dose of gabapentin, or a 150 mg dose of pregabalin orally [1, 6, 7].

After induction, the patient was given dexamethasone 8 mg intravenously and tranexamic acid 10 mg/kg actual body weight for 10 minutes. The intraoperative hemodynamics in this case series were stable. The better hemodynamic stability obtained from regional anesthesia than from general anesthesia is a result of the inhibition of stress hormone release intraoperatively, which leads to a smaller increase and fluctuation in the mean arterial pressure and heart rate. The reduction in blood loss is greater under regional anesthesia because of vasodilation and hypotension as a result of sympathetic blockade. In addition, lower intrathoracic pressure resulting in epidural venous distension also contributes to decreased blood loss and hemodynamic stability. The lower degree of postoperative pain under regional anesthesia may be due to more selective inhibition of regional anesthesia afferent nociceptive sensitivity pathways [8].

After surgery, the patient received analgesic paracetamol (1 g per 6 hours intravenously), gabapentin (300 mg per 24 hours orally), and tramadol (50 mg per 12 hours orally). Postoperative management in the ERAS protocol continues the use of preoperative medications such as nonsteroidal antiinflammatory drugs (NSAIDs) and/or acetaminophen, gabapentin, and possibly, postoperative tramadol [1, 6, 9]. The ERAS protocol for lumbar spine surgery significantly reduces the length of stay (LOS). A comparison between patients who received the ERAS protocol and patients who underwent the same surgical procedure before the initiation of the ERAS protocol (without the ERAS protocol) revealed a significant reduction in postoperative opioid use [10].

The postoperative care component of the ERAS protocol included oral intake and mobilization via early physical therapy during the recovery period within 2 hours of arrival at PACU. Postoperative pain management begins with acetaminophen and NSAIDs. For patients with numerical pain scale (NRS) scores above 4, two doses of tramadol 50 mg were prescribed, and oxycodone 5 mg was prescribed for those with NRS scores above 8. Patients with postoperative nausea and vomiting (PONV) received metoclopramide (10 mg intravenously) or ondansetron (4 mg) treatment, and patients with refractory PONV received scopolamine (1.5 mg transdermally) [1, 6, 9]. An analysis of several studies revealed a decrease in hospital length of stay, a decrease in pain scores, and a decrease in nausea and vomiting in patients under regional anesthesia compared with those under general anesthesia [8]. 

## Conclusion

Regional anesthesia is a safe alternative for lumbar spine surgery that allows for good perioperative hemodynamic stability and significantly reduces opioid requirements to support and apply the concept of ERAS in spinal surgery procedures.

## Data Availability

The authors confirm that the data supporting the findings of this study are available within the article [and/or] its supplementary materials.

## Abbreviations

ASA: American Society of Anesthesiologists
ERAS: Enhanced Recovery After Surgery
kg: Kilograms
LOS: Length of stay
MAP: Mean arterial pressure
Mg: Milligrams
mL: Milliliters
mmHg: Millimeters of mercury
MRI: Magnetic resonance imaging
NSAIDs: Nonsteroidal antiinflammatory drugs
PACU: Postanesthesia Care Unit
PONV: Postoperative nausea and vomiting
